# Improving the Interface Between Resident Doctors and the Quality Improvement and Innovation Team at South West London and St George’s Mental Health NHS Trust– A Quality Improvement Informed Approach

**DOI:** 10.1192/bjo.2026.11432

**Published:** 2026-06-30

**Authors:** Ellen McCloy Smith, Justin Earl, Sarah Galloway, Tammy Osgathorp

**Affiliations:** 1 South West London and St George's Mental Health NHS Trust, London, United Kingdom; 2 Kingston University, London, United Kingdom

## Abstract

**Aims:**

Involvement in Quality Improvement (QI) is an important component of Core Psychiatry Training. The Psychiatry Silver Guide states that trainees should be supported to be involved in one QI project and/or audit per year. We have seen our resident doctors face significant barriers in engaging with QI activities at South West London and St George’s Mental Health NHS Trust (SWLSTG). The Quality Improvement and Innovation (QII) Team coach and track the impact of QI projects at SWLSTG. Since 2023, the QII team have aimed to increase the number of resident doctors involved in QI projects overseen by the QII team.

**Methods:**

Since 2023, we have tested 4 change ideas to increase the number of resident doctors involved in QI projects with oversight from the QII team.

Firstly, a cohort programme was designed to coach a group of resident doctors through a joint project with integrated QI training. The pilot of ‘Streamline QI’ began in early 2023 and included bespoke QI teaching and the coaching of participants through a group project. Subsequent cycles in 2024 and 2025 refined the model.

Additional change ideas tested included creating a SpR Special Interest role to support resident doctors, the targeted advertisement of QI training to doctors and the circulation of a trust criteria for QI project endorsement.

We used registration of QI projects with the QII team and repeated surveys of involvement to measure improvement.

**Results:**

In February 2025, 45 resident doctors were surveyed. 42% of respondents were not involved in QI projects. Of the respondents involved in QI projects, 65% were involved in projects without QII team involvement and 35% had QII oversight. The QII caseload of registered QI projects was reviewed and revealed that 0 resident doctor projects were actively progressing.

In January 2026, a repeat survey of 25 resident doctors showed 40% had not been involved in QI projects. Of the respondents involved in QI projects, 100% had QII oversight. This represents an improvement of 65%. The QII caseload had 8 active resident doctor projects and 9 enrolled in Streamline QI.

Feedback from all three cohorts of Streamline QI has been unanimously positive.

**Conclusion:**

Here we have shown that a multi-dimensional approach can engage resident doctors in QI. Future change ideas to test will include a monthly ‘QI project idea’ clinic for resident doctors and delivering QI training to resident doctors on induction.

